# Students’ view upon graduation: a survey of medical education in Taiwan

**DOI:** 10.1186/1472-6920-12-127

**Published:** 2012-12-22

**Authors:** Wing P Chan, Ting-Yu Wu, Ming-Shium Hsieh, Ting-Ywan Chou, Chih-Shung Wong, Ji-Tseng Fang, Nen-Chung Chang, Chuang-Ye Hong, Chii-Ruey Tzeng

**Affiliations:** 1 Department of Radiology, Wan Fang Hospital, Taipei Medical University, Taipei, Taiwan; 2 Department of Radiology, School of Medicine, College of Medicine, Taipei Medical University, Taipei, Taiwan; 3 Department of Orthopedic Surgery, School of Medicine, College of Medicine, Taipei Medical University, Taipei, Taiwan; 4 Department of Radiology, School of Medicine, Fu-Jen Catholic University, and Cardinal Tien Hospital, Taipei, Taiwan; 5 Department of Anesthesiology, Cathay General Hospital, Taipei, Taiwan; 6 Department of Nephrology, School of Medicine, Chang Gung University, Taipei, Taiwan; 7 Department of Internal Medicine, School of Medicine, College of Medicine, Taipei Medical University, Taipei, Taiwan; 8 Department of Internal Medicine, Wan Fang Hospital, Taipei Medical University, Taipei, Taiwan; 9 Department of Obstetrics and Gynecology, School of Medicine, College of Medicine, Taipei Medical University, Taipei, Taiwan

**Keywords:** Medical education, Questionnaire, Student viewpoint, Survey, Taiwan

## Abstract

**Background:**

Improving the quality of medical education is a key goal of government policy in Taiwan. The aim of this study was to reflect the responses of medical education from the perspective of graduating medical students in Taiwan. This is the first survey study of medical education in Taiwan.

**Methods:**

Using the Medical School Graduation Questionnaire from the Association of American Medical Colleges (AAMC), we distributed 406 questionnaires to medical students of four medical schools in their last semester, and received 270 back (response rate, 66.5%). There were 11 medical schools in Taiwan. Most questions were assessed on a 5-point Likert scale.

**Results:**

Students identified genetics, biochemistry, and ethics as the three most important premedical subjects preparing them for medical education and gross anatomy, physiology, and pharmacology as the three most helpful basic science subjects preparing them for clinical clerkships and electives. Most Taiwanese students were satisfied with their learning experience in internal medicine. Only 55.9% of students were confident that they had acquired the clinical skills required to become a resident, and 70.7% were satisfied with the quality of their medical education.

**Conclusion:**

The study offers preliminary results on the views of graduating students on the medical education system in Taiwan. In particular, our government and medical educators need to continuously put more effort into building students’ confidence in their clinical skills.

## Background

Improving the quality of medical education is a key goal of government policy in Taiwan. The Taiwan Medical Accreditation Council was established in 1991 under the auspices of the National Health Research Institute to ensure better quality of medical education and optimum benefit to patients [1]. In the United States (US), medical school accreditation relies not only on authorization by a government agency but also on medical student input. The Liaison Committee on Medical Education (LCME) (http://www.lcme.org/), which is responsible for developing allopathic medical school policies and guidelines and their assessment and verification in the US, is not a government agency, but is sponsored by the Association of American Medical Colleges (AAMC) and the American Medical Association (AMA). The Department of Education (a federal agency and an independent organization that seeks and utilizes student input and participation) oversees the activities of the LCME [2]. Nationwide surveys of medical students are important tools for examining current education policy and can also be used as a reference by students who intend to attend medical school. However, the results of such surveys have not been previously reported in Taiwan.

Unlike most the US medical schools that required a premedical 4-year degree, admissions in Taiwan only requires completion of a 6-year high school education. Taiwan has a seven-year medical curriculum combining liberal arts and humanities (premedical education) in the first and second years with basic medical sciences in the third and fourth years and clinical sciences in the fifth through seventh years. Fifth- and sixth-year students attend at least a 3-day per week hospital clerkship course. Students obtain relevant clinical knowledge through clinical conferences and practice their communication skills at the bedside. Clerkship students use anatomical models or equipment to practice clinical procedures (e.g., intravenous cannulation, suturing, resuscitation, etc.) and acquire some examination skills (e.g., breast examination, cervical smear, rectal examination, etc.) [3]. In the seventh year, students participate in a full-time internship to complete their clinical training. Interns are trained to perform clinical procedures and examinations in real patients under the supervision or guidance of senior staff.

The AAMC has used its graduation questionnaire for surveying medical students in the US since 1978, and the questionnaire has also been used by many medical schools in other countries [4,5]. Using this questionnaire (in contrast to other serial surveys), student perceptions have been proven to have greater longitudinal stability [6]. The reliability and validity of the questionnaire are, therefore, assured.

The aim of this study was to reflect the responses of medical education from the perspective of graduating medical students in Taiwan, using the AAMC graduation questionnaire. The AAMC graduation questionnaire has two parts: Part I relates to the students’ experiences in their medical schools and Part II is about financial aid and career intentions. To meet the aim of this study, we only used Part I of the questionnaire.

## Methods

The study was approved by the institution review boards of Taipei Medical University, and signed informed consent was obtained from all participants.

We contacted 11 medical school directors in Taiwan by phone or email to determine their willingness to participate in this nationwide survey. Of the 11 schools, two had their own graduation questionnaires and declined to use our forms; three schools had many clinical rotation sites, making collection of the students’ responses difficult; and one school did not reply. In all, five medical schools agreed to participate in the study.

The original Part I survey contained 19 questions. Questions that did not directly relate to the academic experience of medical students, such as questions about student services (Q14, 15, 16, 17), activities that students would like to participate in voluntarily, or their views on being a doctor in Taiwan, were omitted. Finally, 10 questions were selected.

The 10 selected questions were placed into five domains with different content including basic science courses (Q1, 2, 3), clinical experiences (Q4, 5, 13), self-evaluation (Q11, 12), allocation of time for discussion of specific medical issues (Q7), and overall quality of medical education (Q10). The excluded questions were regarded as the sixth domain “others” and are not discussed in this article. The questionnaire was translated into Chinese, approved by professionals, and tested. Between April and July 2009, paper copies of the questionnaire were distributed to medical students during their regular feedback meeting in their last semester of internship.

The questionnaire was designed for medical students in the US. Therefore Question 1 asked about the importance of each premedical education course, and Questions 2 and 3 asked about basic science courses taken after entering medical school. However, the system is different in Taiwan. Students in the first and second year of medical school take liberal arts and humanities courses, which may be the same as premedical courses in the US, and basic science courses are taught in the third and fourth years. Therefore, the questions were re-formulated to fit the situation in Taiwan.

Students were asked their views on medical education, and most responses were evaluated on a five-point Likert scale (ranging from 1, strongly disagree, to 5, strongly agree). In data analysis and for the purposes of discussion, we combined the positive ratings “agree” and “strongly agree” and negative ratings “disagree” and “strongly disagree.” “No opinion” was omitted, and missing data for each question were not included. Data were analyzed using SPSS version 18.0 software (SPSS Inc, Chicago, IL).

## Results

Data for the five selected domains are given as percentages to provide an overview of the result. One of the five participating schools was excluded because the response rate was lower than 5%. Consequently, data from four out of 11 medical schools (three private and one public) were obtained (Table 1). A total of 406 copies of the questionnaire were distributed, and 270 copies were returned. The response rate was 66.5%. At the time of this survey, 85.5% of students were aged 24 to 26 years, 100% of students had finished their internship courses in at least two of the four major disciplines (internal medicine, surgery, obstetrics and gynecology, and pediatrics), and 75.5% of students had finished their internship in all four major disciplines. 

**Table 1:** Demographic data of respondents to the questionnaire at four medical schools in Taiwan

**School**	**Male (%)**	**Female (%)**	**Missing (%)**	**Respondents (n)**	**Total number of surveys distributed (n)**	**Response rate (%)**
**A**	27 (43.5%)	20 (32.3%)	15 (24.2%)	62	150	41.3
**B**	11 (29.7%)	14 (37.8%)	12 (32.5%)	37	42	88.1
**C**	49 (57.6%)	19 (22.4%)	17 (20.0%)	85	100	85.0
**D**	51 (59.3%)	20 (23.3%)	15 (17.4%)	86	114	75.4
**Total**	138 (51.1%)	73 (27.0%)	59 (21.9%)	270	406	66.5

### Premedical education and basic science courses

As for the importance of liberal arts and humanities courses to the education of medical students, genetics and biochemistry were considered important or very important, respectively, by 47.4% and 44.1% of students and ethics was considered important by 41.2% of students.

The basic science courses that students thought were most helpful for clinical work before entering their clerkship were gross anatomy (90.0%), physiology (88.9%), and pharmacology (87.8%). When asked about their experience during basic science courses, 61.1% of the respondents believed that the content objectives of these courses had been made clear to them, and 60.4% considered that the content objectives in this phase matched the content of the examination. Of the respondents, 21.1% disagreed or strongly disagreed that the basic science course content had sufficient illustrations of clinical relevance (Table 2). 

**Table 2:** Basic Science Courses: Based on your experiences, indicate whether you agree or disagree with the following statements about medical school?

**Item**	**Results (%)**							
	**Strongly agree**	**Agree**	**No opinion**	**Disagree**	**Strongly disagree**	**Missing rate**	**Mean**	**Count**
**1**	8.9	52.2	20.4	13.7	1.9	2.9	3.4	262
**2**	8.9	51.5	23.0	11.9	1.9	2.8	3.5	262
**3**	8.9	49.6	21.5	12.6	4.8	2.6	3.4	263
**4**	9.6	48.9	21.9	14.1	2.6	2.9	3.4	262
**5**	9.3	44.8	22.2	17.8	3.3	2.6	3.3	263

Key to statement number:

1. Basic science content objectives were made clear to students.

2. Basic science content objectives and examination content matched closely.

3. Basic science content provided relevant preparation for clerkship.

4. Basic science content was sufficiently integrated.

5. Basic science content illustrated clinical relevance sufficiently.

### Clinical experience

Most of the respondents (76.2%) agreed that internal medicine provided them with a good learning experience, but fewer students were satisfied with their learning experience in family medicine (43.0%) (Table 3). Most (78.5%) agreed that the clinical internship year was important for enhancing their medical education. Also, 77.8% believed that the last year of medical education was helpful in their preparation for residency (Table 4). 

**Table 3:** Clinical experiences: rate the quality of your educational experiences in the following clinical clerkships

**Item**	**Results (%)**							
	**Very good**	**Good**	**Poor**	**Very Poor**	**Not applicable**	**Missing rate**	**Mean**	**Count**
**1**	18.1	58.1	19.3	2.2	0	2.3	3.9	264
**2**	17.8	55.6	21.5	2.6	0	2.5	3.8	263
**3**	15.2	54.8	24.4	3.0	0.4	2.2	3.7	264
**4**	13.7	52.2	28.5	2.6	0.7	2.3	3.7	264
**5**	14.1	51.5	29.3	2.2	0.7	2.2	3.7	264
**6**	15.6	48.1	26.7	1.5	5.9	2.2	3.6	264
**7**	14.4	45.9	27.4	3.3	6.3	2.7	3.5	263
**8**	10.0	48.9	33.0	1.9	4.1	2.1	3.5	264
**9**	6.7	36.3	38.5	6.7	9.6	2.2	3.2	264

Key to statement number:

1. Internal medicine.

2. Pediatrics.

3. Obstetrics and gynecology.

4. Surgery.

5. Neurology.

6. Psychiatry.

7. Emergency medicine.

8. Radiology.

9. Family medicine.

**Table 4:** Clinical experiences: indicate whether you agree or disagree with the following statements about your final year of medical education

**Item**	**Results (%)**							
	**Strongly agree**	**Agree**	**No opinion**	**Disagree**	**Strongly disagree**	**Missing rate**	**Mean**	**Count**
**1**	32.2	46.3	13.7	1.9	0.7	5.2	3.9	256
**2**	27.8	50.0	13.0	3.0	1.1	5.1	3.9	256
**3**	12.2	58.9	19.6	3.3	0.7	5.3	3.6	256
**4**	11.9	56.3	20.4	5.6	0.7	5.1	3.6	256
**5**	7.8	30.0	34.8	18.5	3.3	5.6	3.0	255

Key to statement number:

1. The final year (internship in Taiwan) was important for enhancing my medical education.

2. The final year was helpful in my preparations for residency.

3. I received appropriate guidance in the selection of sixth year (Taiwan) elective activities.

4. At my school, time for elective activity in the sixth year (Taiwan) was adequate.

5. Additional required activities should be added to the final year at my medical school.

### Allocation of time to specific medical topics

One-fourth of the respondents (25.9%) regarded the time devoted to population-based medicine as not enough (Table 5). The largest percentage of respondents (34.4%) thought that instruction in health surveillance strategies (a topic in population-based medicine) was inadequate (Table 6) (although this was not the majority of the respondents, owing to the large number of missing data), and 33.7% of respondents thought that instruction in biomedical, chemical, and natural disaster management was inadequate. 

**Table 5:** Time allocation to specific medical issues: do you believe that your instruction in the following areas was inadequate, appropriate, or excessive?

**Item**	**Results (%)**				
	**Adequate**	**Inadequate**	**Excessive**	**Missing rate**	**Count**
**1**	62.9	25.9	0.6	10.6	241
**2**	72.4	21.3	1.2	5.1	256
**3**	74.4	19.5	0.8	5.3	256
**4**	81.6	16.8	1.6	0.0	270
**5**	83.8	10.9	0.4	4.9	257

Key to statement number:

1. Population based medicine.

2. Practice of medicine.

3. Other medical topics.

4. Evidence based medicine.

5. Clinical decision making and clinical care.

**Table 6:** Do you believe that your instruction in the following areas of population based medicine was inadequate, appropriate, or excessive?

**Item**	**Result (%)**				
	**Adequate**	**Inadequate**	**Excessive**	**Missing rate**	**Count**
**1**	58.8	34.4	0.7	6.4	253
**2**	59.6	33.7	0.4	6.3	256
**3**	59.6	33	0.7	6.7	252
**4**	60.0	32.2	1.5	6.3	256
**5**	62.6	30.4	0.4	6.6	252
**6**	65.2	28.1	0.4	6.3	256
**7**	66.7	27.0	0.4	5.9	254
**8**	66.3	27.0	0.7	6.0	254
**9**	67.8	25.6	0.4	6.2	253
**10**	68.5	25.2	0.4	5.9	254
**11**	68.5	24.4	0.7	6.4	253
**12**	71.1	22.2	0.4	6.3	256
**13**	71.5	21.9	0.4	6.2	253
**14**	73.3	20.0	0.4	6.3	256
**15**	73.3	19.6	0.4	6.7	252
**16**	74.4	17.8	0.7	7.1	251
**17**	75.6	17.0	0.7	6.7	252

Key to statement number:

1. Health surveillance strategies.

2. Biomedical, chemical, and natural disaster management.

3. Culturally appropriate care for diverse populations.

4. Health services financing.

5. Health and healthcare disparities.

6. Occupational medicine.

7. Health policy.

8. Global health issues.

9. Public health.

10. Biostatistics.

11. Environmental health.

12. Role of community health and social service.

13. Community medicine.

14. Women’s health.

15. Epidemiology.

16. Health determinants.

17. Disease prevention.

### Self-evaluation

The graduate questionnaire dealt with three self-evaluative aspects: technology skills, communication skills, and student’s readiness for residency. In all, 84.1%, 80.0%, and 48.9% of respondents agreed that they could, respectively, use a computer-based clinical record-keeping program to find and keep records of patient information, carry out necessary sophisticated searches of an information database, and use telemedicine (Table 7). 

**Table 7:** Self-evaluation of technology skills: indicate your level of agreement with the following statements: I am confident that I have the appropriate knowledge and skills to:

**Item**	**Results (%)**							
	**Strongly agree**	**Agree**	**No opinion**	**Disagree**	**Strongly disagree**	**Missing rate**	**Mean**	**Count**
**1**	16.3	67.8	8.9	1.5	0.4	5.1	3.8	256
**2**	13.3	66.7	13.3	1.5	0	5.2	3.8	256
**3**	13.7	65.2	14.1	1.5	0.4	5.1	3.8	256
**4**	8.1	52.2	26.3	7.0	1.1	5.3	3.4	256
**5**	8.9	40.0	27.0	14.8	4.1	5.2	3.2	256
**6**	8.9	40.7	32.6	10.7	1.9	5.2	3.3	256

Key to statement number:

1. Use a computer-based clinical record keeping program, both for finding and recording patient-specific information.

2. Carry out necessary sophisticated searches of medical information databases.

3. Protect the confidentiality of private information obtained from patients and colleagues when the information is stored on a computer.

4. Critically review published research.

5. Use telemedicine.

6. Use point of care technology for clinical purposes.

Regarding communication skills, 79.6% were comfortable in assessing the health practices of a patient using alternative therapies, 76.0% were able to negotiate with a patient who requests unnecessary tests or procedures, and 46.3% were comfortable in discussing with the patient a prescription error that they had made (Table 8). 

**Table 8:** Self-evaluation of communication skills: indicate your level of agreement with the following statements: I am confident that I have the appropriate knowledge and skills to:

**Item**	**Results (%)**							
	**Strongly agree**	**Agree**	**No opinion**	**Disagree**	**Strongly disagree**	**Missing rate**	**Mean**	**Count**
**1**	12.6	67.0	12.6	2.6	0	5.2	3.7	256
**2**	14.1	61.9	14.4	3.7	0.7	5.2	3.7	256
**3**	11.1	50.0	20.7	10.7	2.2	5.3	3.4	256
**4**	9.3	51.1	23.0	10.7	0.7	5.2	3.4	256
**5**	7.0	39.3	25.9	20.4	2.2	5.2	3.1	256
**6**	8.9	45.2	25.2	13.7	1.9	5.1	3.3	256

Key to statement number:

1. Assess the health practices of a patient using alternative therapies.

2. Negotiate with a patient who is requesting unnecessary tests or procedures.

3. Provide safe sex counseling to a patient whose sexual orientation differs from mine.

4. Discuss treatment options with a patient with terminal illness.

5. Discuss a prescription error I made with the patient.

6. Initiate discussion of DNR (“do not resuscitate”) orders with a patient or family member.

When preparedness for beginning a residency program was considered, 77.4% thought that they understood the ethical and professional values that were expected of the profession, but only 55.9% were confident that they had acquired clinical skills required to become a resident (Table 9). The average point score was lower for “I am confident that I have acquired the clinical skills required to begin a residency program” than for other items. 

**Table 9:** Self-evaluation of preparedness for residency program: indicate whether you agree or disagree with the following statements about your preparedness for beginning a residency program

**Item**	**Results (%)**							
	**Strongly agree**	**Agree**	**No opinion**	**Disagree**	**Strongly disagree**	**Missing rate**	**Mean**	**Count**
**1**	13.0	64.4	14.4	2.2	0.7	5.3	3.7	256
**2**	10.0	56.7	22.6	4.8	0	5.9	3.5	254
**3**	10.0	63.0	18.5	3.3	0	5.2	3.6	256
**4**	11.5	59.6	20.0	3.7	0	5.2	3.6	256
**5**	9.3	59.3	21.1	4.8	0.4	5.1	3.6	256
**6**	13.7	54.8	21.1	4.4	0.4	5.6	3.6	255
**7**	8.1	47.8	24.1	13.7	1.1	5.2	3.3	256

Key to statement number:

1. I understand the ethical and professional values that are expected of the profession.

2. I have basic skills in clinical decision making and the application of evidence based information to medical practice.

3. I have the fundamental understanding of the basic disease mechanisms, clinical presentation, and principles of diagnosis and management for the common conditions encountered in the major clinical disciplines.

4. I have the communication skills necessary to interact with patients and health professionals.

5. I have a fundamental understanding of the issues in social sciences of medicine (e.g., ethics, humanism, professionalism, organization and structure of the health care system).

6. I believe I am adequately prepared to care for patients from diverse backgrounds.

7. I am confident that I have acquired the clinical skills required to begin a residency program.

### Overall satisfaction

Of the 270 respondents, 70.7% indicated satisfaction (61.1% agreed and 9.6% strongly agreed) with the overall quality of their medical education, while 6.3% reported dissatisfaction (5.6% disagreed and 0.7% strongly disagreed), with 17% expressing no opinion.

## Discussion

Our survey was conducted in 2009, and therefore, we compared our data with data from the 2009 study of US graduates [7], except the item of ‘ethics’ was compared with AAMC 2008 because the item was removed from the table of 2009 graduation questionnaire. The percentage of students who believed that ethics was an important undergraduate medical education course taken in their first and second year of medical school was higher in Taiwan (41.2%) than in the US (33.3%) [8]. According to Lehmann et al. [9], most schools agree that courses in ethics should be mandatory for all students (94%). In Taiwan, medical ethics has been part of the undergraduate medical education course since 1993 [10], and is regarded as critical for improving the quality of medical care and reducing the tension inherent in the doctor-patient relationship. Our survey (15 years after implementation of ethics education) estimates about two fifths of the Taiwanese students believe that ethics is an important preclinical course. This view conveys the message that competence in medical ethics is central to being a doctor.

Medical students in both Taiwan and the US considered genetics (47.4% and 65.4%, respectively) and biochemistry (44.1% and 68.0%, respectively) to be the most important subjects (AAMC 2009 Report). As the least important subject, physics was identified by Taiwanese students (59.6%) and organic chemistry by US students (52.5%). Our students generally viewed organic chemistry to be of some value, but responded that biochemistry was of greater utility in medical school.

The basic science subjects selected by Taiwanese and US students as helpful for successful completion of clinical rotations were similar. The top three subjects were gross anatomy (90%), physiology (88.9%), and pharmacology (87.8%) in Taiwan, and pathophysiology of disease (88.4%), gross anatomy (86.7%) and physiology (86.2%) in the US. Flexner concluded that the basic sciences of anatomy, physiology, biochemistry, pathology, and pharmacology are fundamental to medical practice; a similar view by students was noted in our study [11].

Fewer Taiwanese students than US students agreed that basic science subjects offered clear learning objectives (60.4% vs. 85.0%). Approximately one fifth of Taiwanese and US students, respectively, responded that the basic science curriculum was not fully integrated into the clinical practice curriculum. Although mathematics courses have been traditionally thought of as promoting logical and analytical thinking, respondents conveyed little enthusiasm for it as a prerequisite.

Our students’ views on clinical relevance are similar to those of US students. Over the past century, the core structure (two years of science followed by two years of clinical apprenticeship) and processes (instruction through lectures and small group sessions, followed by attachment to diverse clinical rotations in diverse settings) remains unchanged for most medical schools in the US. In the past few years, our medical educational system has gradually been transformed from a discipline-based curriculum to a block system (organ-based) curriculum, which integrates normal structure and function with the pathophysiology of disease [12]. Problem-based learning taught by clinicians has been successfully implemented to facilitate integration of clinical and basic science teaching in the preclinical curriculum nationwide, and clinical vignettes have been successfully used as test items for preclinical students to reinforce relevant basic science concepts and enhance interest.

Although medical educational systems differ between countries, the rate of satisfaction with the respective medical programs is similar (86.6% of American and 86.3% of Australian students [7,13] compared with 70.7% Taiwanese students). More importantly, the survey of self-confidence about beginning a residency program revealed that 88.6% of American students and only 55.9% of Taiwanese students thought they had the clinical skills necessary to perform as a resident. The results of our study indicate that the clinical training of Taiwanese medical students is insufficient. Early medical education in Taiwan puts greater emphasis on lectures and academic knowledge than on clinical training. The Objective Structured Clinical Examination (OSCE) formally became part of the medical education process in Taiwan in 2006 and part of the licensure test in 2012 [14,15]. By contrast, the US has been advocating clinical education since the early 20^th^ century. The Flexner report established the basic structure of US medical education, which values both foundational science and clinical application as necessary for doctor training [11]. For example, the second part of the three-step United States Medical Licensing Examination [16] is the clinical skills examination. In this part of the examination, the student is required to examine a patient, show basic assessment skills, develop a preliminary diagnosis, and document his interaction with the patient. By hands-on testing, the student’s clinical skills can be more closely assessed.

Results of our study also may indicate some cultural differences between our students and their American counterparts. It could be inferred that Taiwanese students are less inclined to evaluate themselves in an excessively positive manner [17].

There are a few limitations to the present study. Although the response rate of the survey was not low (66.5%), only four of 11 medical schools participated in this study. Therefore, the sample of schools might not be representative of the nationwide reality. Comparison of our results and those of the US may be biased, as their medical educational systems are quite different from ours. The teaching of various disciplines may vary among the four medical schools surveyed, but variation is minor, as the system of medical education is uniform throughout our country.

## Conclusion

In summary, the study offers preliminary results on the views of graduating students on the medical education system in Taiwan. In particular, our government and medical educators need to continuously put more effort into building students’ confidence in their clinical skills.

## Competing interests

The authors declare that they have no competing interests.

## Authors’ information

WPC is Professor of Radiology, School of Medicine, and Associate Dean, College of Medicine, Taipei Medical University, and Chief of Radiology, Wan Fang Hospital, Taipei Medical University. TYW is Research Assistant, Department of Radiology, School of Medicine, Taipei Medical University. MSH is Professor and Chief, Department of Orthopedic Surgery, Taipei Medical University Hospital, and Director, School of Medicine, Taipei Medical University. TYC is Associate Professor of Radiology, School of Medicine, Fu-Jen Catholic University, and Chief of Radiology, Cardinal Tien Hospital. CSW is Professor, School of Medicine, National Defense Medical Center, and Attending Staff, Department of Anesthesia, Cathay General Hospital. JTF is Professor of Nephrology, School of Medicine, Chang Gung University, and Director, School of Medicine, Chang Gung University. NCC is Professor and Chief, Department of Internal Medicine, Taipei Medical University, and Associate Dean, College of Medicine, Taipei Medical University. CRT is Professor and Chief, Department of Obstetrics and Gynecology, School of Medicine, Taipei Medical University, and Dean, College of Medicine, Taipei Medical University. CYH is Professor, Department of Internal Medicine, School of Medicine, Taipei Medical University, and Attending Staff, Wan Fang Hospital, Taipei Medical University.

## Authors’ contributions

WPC led on conception, design, interpretation of data, reviewing of literatures, revising the article and critically appraising the content, and is the guarantor of the paper. TYW contributed to review literatures, collection of data, and drafting of the article. MSH, TYC, CSW, JTF, NCC, CRT were responsible for collection of data and interpretation of data. CYH contributed to interpret the data, revising the article and critically appraising the content. All authors have approved the final version of the article submitted.

## Pre-publication history

The pre-publication history for this paper can be accessed here:

http://www.biomedcentral.com/1472-6920/12/127/prepub
